# Future Perspectives of ERAS: A Narrative Review on the New Applications of an Established Approach

**DOI:** 10.1155/2016/3561249

**Published:** 2016-07-18

**Authors:** Dario Bugada, Valentina Bellini, Andrea Fanelli, Maurizio Marchesini, Christian Compagnone, Marco Baciarello, Massimo Allegri, Guido Fanelli

**Affiliations:** ^1^2^a^ Anestesia Anestesia, Rianimazione e Terapia del Dolore, Azienda Ospedaliero Universitaria, Via Gramsci 14, 43126 Parma, Italy; ^2^Department of Surgical Sciences, University of Parma, Via Gramsci 14, 43126 Parma, Italy; ^3^SIMPAR Group (Study in Multidisciplinary Pain Research), Italy; ^4^U.O. Anestesia e Terapia Intensiva Polivalente, Azienda Ospedaliero-Universitaria Policlinico S. Orsola-Malpighi, Via Albertoni 15, 40138 Bologna, Italy

## Abstract

ERAS approach (Enhanced Recovery After Surgery) is a multimodal, perioperative pathway designed to achieve early recovery after surgery. ERAS has shown documented efficacy in elective surgery, and the concept of “multimodal” and “multidisciplinary” approach seems still to be of higher importance than each single item within ERAS protocols. New perspectives include the use of ERAS in emergency surgery, where efficacy and safety on outcome have been documented, and flexibility of traditional items may add benefits for traditionally high-risk patients. Obstetric surgery, as well, may open wide horizons for future research, since extremely poor data are currently available, and ERAS benefits may translate even on the baby. Finally, the concept of “outcome” may be extended when considering the specific setting of cancer surgery, in which variables like cancer recurrence, early access to adjuvant therapies, and, finally, long-term survival are as important as the reduced perioperative complications. In this perspective, different items within ERAS protocols should be reinterpreted and eventually integrated towards “protective” techniques, to develop cancer-specific ERAS approaches keeping pace with the specific aims of oncologic surgery.

## 1. The Concept of ERAS

ERAS approach (Enhanced Recovery After Surgery) is a multimodal, perioperative care pathway designed to achieve early recovery after surgery [1]. The concept was developed in the 90s and was enriched with new elements over years. ERAS items encompass anesthesia/analgesia, goal-direct fluid therapy, prevention of nausea and ileus, thromboembolic prophylaxis, minimally invasive techniques, temperature monitoring, early nutrition, and early mobilization [2]. A main reason for the effectiveness of the ERAS protocols relies on the ability of each element within protocols to reduce stress response to injury and maintain homoeostasis [3]. Common to all ERAS programs is the attempt to reduce the surgical stress response to surgery and decrease perioperative complications; the final result is to speed up patient's recovery, reducing health costs [4].

*Surgical stress response* (SSR) is represented by hormonal and metabolic changes resulting in immune and endocrine responses, proportionally with the extent of surgical tissue lesion. Local changes impact the inflammatory reaction in the whole body, leading to widespread effects on organ functions and on the development of complications. SSR plays a major role in respiratory, cardiac, and thromboembolic complications, resulting in a general catabolic state and in strong immune-depressant effects [3]. Preventing SSR is the key mechanism on which ERAS perioperative programs are based [5].

Because of evidence on the positive effects of this approach, ERAS has now extended to several types of surgeries. In this narrative review, we want to present the newest field of applications of this philosophy. Despite the fact that several possible topics may be discussed and much progress is still possible within ERAS, we decided to focus only on the most innovative ones (even when few studies exist), that is, those that are supposed to have the higher development in the future years and produce some changes in the philosophy of ERAS towards a patient-specific (and not only surgery-specific) approach. The latest applications in emergency and obstetric surgery are presented, as well as implications in the specific subset of cancer patients, in which other “outcomes” may add to the traditional ones and may promote the development of cancer-specific ERAS protocols in the future.

## 2. The Proven Efficacy of ERAS: A History of Success

The benefit and safety of ERAS protocol have been validated in different studies, and the validity of ERAS approach is no more questioned. A specific society entitled to the ERAS approach exists, including the most important experts in the field and producing procedure specific guidelines easily accessible on the web and able to guide any perioperative physician who wants to develop an ERAS approach within his/her institution [1]. Guidelines include urologic [6], gastric [7], biliary and pancreatic [8], colon [9], rectal [10], gynecologic [1, 11], and bariatric surgery [12]. All guidelines are intended to reduce surgical stress and complications, thus speeding up recovery and reducing hospital stay.

Despite the amount of strategies included in ERAS protocols, introduction in clinical practice resulted to be feasible in different realities, and a recent study reported that the rate of compliance to each ERAS item within a protocol correlates with the length of the hospital stay (the higher the compliance, the shorter the length of stay) [13].

Authors who have historically conceived and validated ERAS protocols have already suggested key points for the development of successful ERAS programs, which are based on knowledge of institutional reality, on continuous communication within the multidisciplinary team, and on application of common protocols for patients care [14].

## 3. ERAS: New Perspectives of Application

### 3.1. Emergency Surgery

In contrast to what happened for elective surgery, there has been little improvement in outcomes or morbidity for patients undergoing emergency procedures; emergency surgical operations carry a mortality rate at least ten times higher than many similar elective procedures [15] and ERAS strategies may be of even greater advantage in such kind of setting.

Unfortunately, few studies investigated the use of ERAS model in emergency surgery. The setting of emergency obviously limits compliance with the entire protocol, since this was initially designed for elective situations. Moreover, emergency patients might be considered at higher risk [16]. However, the potential benefits of those evidence-based protocols should not be denied to patients undergoing urgent surgery [17]. The flexibility of the program is crucial for the effective application in this area. Some items within ERAS protocols for abdominal surgery will be difficult to apply in relation to the more severe patient conditions and to the unplanned hospitalization, particularly in the preoperative setting [17].

Despite this, Roulin et al. have studied the application of ERAS in urgent colectomy, defined as “*colonic resection performed during an unplanned hospital admission*”; they have demonstrated that many of the ERAS items can be applied for such situation, and no significant adverse effect was observed despite higher American Society of Anesthesiologists (ASA) status, Portsmouth-POSSUM (P-POSSUM) scores, and more stressful procedures in urgent patients [17].

Another study evaluated the safety and efficacy of ERAS approach on patients undergoing laparoscopic repair for perforated peptic ulcer. ERAS resulted in improved surgical outcome by reducing the occurrence of postoperative ileus; the authors suggested this result to be associated with the lack of routine nasogastric decompression and with early oral feeding. The use of nonsteroidal anti-inflammatory drugs for postoperative pain seemed to provide beneficial effects, as well [18].

In conclusion, although studies on ERAS in emergency surgery are rare, early results account for the flexibility, effectiveness, and safety of this approach. Further studies are needed to confirm such evidence in wider range of surgical procedures, in the perspective of developing patient-related, surgery-specific ERAS approaches for emergency procedures (as previously done for elective pathways).

### 3.2. Obstetric Surgery

Cesarean section (CS) is an interesting field of research for ERAS, whereas the return to normal function is more precious than ever in this type of patients. The need to standardize ERAS protocols for parturient undergoing CS is perceived by the research and clinical community. First experiences in this field have been starting in the last years. A recent study has shown that the application of the ERAS protocol resulted in a substantial increase in the proportion of women leaving hospital the day after elective CS, without increasing the rates of readmission [19]. Otherwise, peculiar characteristics of patient's management within the institution where the study was held limit the applicability to common clinical practice worldwide: in that hospital, it is routine practice for all women to be discharged following CS and be visited at home by a community midwife, allowing ready access to a health professional in case of problems arising after discharge. This is the first study on the topic, and further experiences are needed to evaluate feasibility, effectiveness, and safety (and to eventually standardize any approach), but CS and obstetrics are interesting field of research for ERAS, which may have influence even on the baby's outcome.

## 4. Cancer Surgery: Do Specific Outcomes Imply Specific Approaches?

Even with the best surgical technique, surgery for cancer is associated with release of tumor cells [20].

Several perioperative factors contribute to the development of remote metastasis [21], but since the anticancer immune response is a primary determinant of cancer progression, it seems straightforward to hypothesize that interventions aimed at reducing exposure to immunosuppressive factors would improve patient outcomes after potentially curative cancer resection [20]. Some specific considerations are needed for cancer patients.

### 4.1. Protection from Stress and Analgesia in Cancer Patients: Epidural or Not?

Adequate pain control for the entire perioperative period is mandatory in ERAS protocols and is even more required in the perspective of oncological outcome. Indeed, pain activates the hypothalamic-pituitary axis and the sympathetic system, giving immunosuppression [21]. Authors suggest that the management of perioperative pain is a critical factor in preventing surgery-induced decreases in host resistance against metastasis in animal study [22].

Thus, pain control is the main target, but the choice in the analgesic strategy is also crucial, since the benefit of pain control may be overcome by tumor promoting effect of some drugs. The opioid mu-receptor agonists commonly used for anesthesia and perioperative analgesia are immune-suppressive: morphine and fentanyl suppress the innate and acquired immunity [21], and opioids may impair host defense against circulating tumor cells after surgery [23]. Morphine also has effects on angiogenesis and on progression of cancer [24]: in a recent paper, morphine at clinical relevant doses enhanced microvessel formation and increased breast cancer progression [25], while a higher density of peritumoral lymphatics was associated with morphine in animal models [24].

Nevertheless, in vitro studies gave conflicting results on the topic. Long-term morphine treatment reduces leukocyte migration and recruitment to reduce angiogenesis and tumor growth in long-term cultures of mouse Lewis lung carcinoma cells [26]. In another study, Willner et al. found that short-term exposure to morphine inhibits neural progenitor cell (NPC) proliferation, promotes apoptosis, and alters NPC differentiation of isolated prenatal cerebral cortical cells in vitro [27].

Although there is no definitive evidence that opioids worsen outcomes in cancer patients [28], opioid-sparing strategies are of great interest for cancer surgery and have gained much attention in the literature. Also, opioid-sparing strategies have been pursued for years in ERAS protocols, becoming one of the cornerstones for enhanced recovery. Thus, the avoidance/reduction of opioids and the maintenance of immune homeostasis are common target of ERAS protocols and cancer surgery and regional anesthesia/multimodal analgesia play an important role in both cases [29]. Regional anesthesia is a key element of most of ERAS protocols [30], with epidural being considered the gold-standard technique to provide prompt recovery.

Despite this evidence, the role of epidural anesthesia (EA) has been questioned [31] because of the risk-benefit ratio and the availability of new alternative technique for analgesia; some experts have stressed the need for new studies investigating alternative analgesic strategies in the optics of ERAS approaches [30]. Techniques like TAP block, rectus sheath block, PECS block, and continuous wound infusion may be beneficial and likely provide comparable or even faster recovery compared to EA.

Otherwise, no studies have investigated any protective effect on SSR using such techniques instead of epidural. EA has the advantage of reducing the sympathetic stimulation and opioid consumption; EA reduces cortisol [32] and epinephrine levels and leads to increased level of insulin [33] and seems also to have beneficial effects on immunological response [34]. This protective effect is supposed to be limited without EA, since most of the new techniques provide only parietal analgesia (no visceral components). Further, concerns exist about timing: some blocks have limited evidence as continuous technique, and continuous infusion of local anesthetics within the surgical wound covers only the postoperative period.

Regarding the potentially limited effect on SSR and considering the concomitant use of rescue opiates in the postoperative period, the efficacy of such new techniques may be questioned in the specific setting of cancer patients, where main outcome variables include cancer spread and recurrence, access to adjuvant therapies, and, finally, long-term survival. In this perspective, specific cancer-oriented, epidural-based ERAS approaches may be adopted, differently from noncancer patients (in whom neuraxial anesthesia should be probably replaced by less invasive techniques).

In this perspective, some authors have already suggested a protective role of regional anesthesia on cancer recurrence [35]. The use of EA seems to reduce tumor progression and increase oncological outcome in different tumors [36], but evidence is limited by methodological bias and by the retrospective nature of studies [37].

Finally, a limiting factor in cancer outcome is* Persistent Postsurgical Pain* (PPSP), which may condition long-term opioid therapies and more difficult access to life-saving therapies (radiotherapy and chemotherapy).

Again, key elements of ERAS protocols are part of the so-called “protective anesthesia,” an innovative anesthetic regimen [38] that relies on completely opioid-free, antihyperalgesic techniques (including strong neuraxial block) aimed at reducing the impact of hyperalgesia and limiting the development of central sensitization and PPSP. Such method is based on multimodal analgesia and encompasses drugs that have common effects on pain pathways and cancer cells (nonsteroidal anti-inflammatory drugs, lidocaine iv infusion) [29]. No studies exist on the effects of such protocols on cancer outcome and PPSP development (and consequences) on cancer patient, but future studies may investigate the use of such opioid-free strategies for the development of cancer-specific ERAS protocols (combining enhanced recovery and improved cancer outcome).

### 4.2. PONV and Dexamethasone

Prevention and treatment of postoperative nausea and vomiting (PONV) are another important component of ERAS protocols, as PONV may result in retardation of enteral feeding. Early nutrition is a major component of ERAS protocol and adds benefits in maintaining metabolic homeostasis [3]: it has a positive impact on nutritional status, on the immune system, and on speeding intestinal recovery in patients with gastric cancer [39].

Dexamethasone is one of the most widely prescribed drugs against PONV, also considering its low cost; there is plenty of evidence on the efficacy of dexamethasone, which is supposed to act through central and peripheral mechanisms: inhibition of prostaglandins, production of anti-inflammatory factors, and decreased production of endogenous opioids [40]. The effective dose as antiemetic use corresponds to 2.5–10 mg daily [40]. A recent review, however, showed that 4 mg to 5 mg of dexamethasone for prevention of PONV appears to have similar effects as 8–10 mg when the drug is used alone or in combination [41].

Otherwise, concerns still exist on wound healing and on the immune-depressant effect of dexamethasone in the context of cancer surgery; a recent study recommends prudence in pharmacological decisions, despite the fact that any association was demonstrated between the use of dexamethasone and the increase in surgical site infections [42].

Few studies have addressed the topic of cancer outcome and use of perioperative dexamethasone, and results are conflicting. In patients affected by rectal cancer, a better three-year survival was noticed in the untreated group compared with patients given dexamethasone perioperatively [43]. In another study, the use of dexamethasone is not associated with an increased risk of endometrial cancer disease relapse [44]. Because of the paucity of studies addressing this issue, no definitive conclusions can be made about the topic, but considering the potential detrimental effects of dexamethasone on long-term cancer outcome, caution should be kept in the choice of this treatment in the specific setting of cancer surgery.

## 5. Laparoscopic Surgery and ERAS: All for One, One for All

A key element in ERAS approaches is the use of minimally invasive surgery. Video-Laparoscopic Surgery (VLS) has been shown to determine lower stress response and fewer complications and to reduce hospital stay and mortality [45] and is currently considered the gold standard for many types of intervention. VLS reduces neurohumoral activation that adversely affects recovery [3, 46] by minimizing the access wound and the release of postoperative inflammatory factors. Although the use of VLS is well appreciated in ERAS programs, the need to introduce ERAS approach in VLS procedures has been debated; some authors so have wondered whether the introduction of the ERAS protocol in laparoscopic surgery would bring additional advantages.

A meta-analysis of the six studies concluded that VLS + ERAS is a more reliable treatment for colorectal malignancy, compared with laparoscopic conventional care treatment. VLS + ERAS reduces complications while carrying similar risks of anastomotic leakage, wound infection, obstruction, and readmission [47]. Other studies have also confirmed the effectiveness of the ERAS protocol in laparoscopic surgery; relevant benefits include reduced time of hospitalization and reduced complications [48]. A trial encompassing all care pathways (i.e., comparing 400 patients randomized to receive either VLS + ERAS, VLS + conventional care, open surgery + ERAS, or open surgery + conventional care) confirmed that optimal perioperative treatment for patients requiring segmental colectomy for colon cancer is laparoscopic resection embedded in a fast-track program [49]. Thus, merging ERAS protocols and VLS seems to offer further advantages than those offered by VLS itself, and ERAS should be probably adopted regardless of the surgical technique. This observation confirms the concept of “multimodality” as the base of ERAS success, that is, that the combination of the different items included in ERAS protocols is responsible for enhanced rehabilitation rather than a single element itself.

Joining ERAS and VLS seems even more crucial in cancer patients. As mentioned above, VLS results in a lower stress response (smaller rise of proinflammatory cytokines and of concentration of C-reactive protein [50, 51]), reducing immune impairment and likely improving host's defense against cancer. A study showed that laparoscopic surgery for primary tumor in HCC patients determines not only a lower inflammatory response but also a reduction of circulating tumor cells (CTCs) [52]. Patients undergoing laparoscopic surgery exhibited a trend towards shorter time intervals to start adjuvant chemotherapy after colorectal oncological surgery [53], and the study by Gustafsson et al. [54] reported an improved 5-year cancer-specific survival after colorectal cancer surgery in patients with higher adherence to ERAS items (>70%). As the authors reported, an important role may be played by the decreased stress response achieved through the application of ERAS protocol.

## 6. Conclusions

In conclusion, the ERAS approach in abdominal surgery has shown documented efficacy in elective surgery, and the concept of “multimodal” and “multidisciplinary” approach seems still to be of higher importance than the single items themselves. New perspectives include the use of ERAS in emergency surgery, where the first studies were able to demonstrate flexibility and to bring benefits in outcome, and obstetric surgery, where a whole world of possibilities to be explored exists, since extremely poor data are currently available. Finally, it would be important to extend the ERAS outcomes beyond the perioperative period in oncological surgery, analyzing the different components of the protocol to develop cancer-specific ERAS approaches.
